# Systematic review to assess the possibility of return of cerebral and cardiac activity after normothermic regional perfusion for donors after circulatory death

**DOI:** 10.1002/bjs.11046

**Published:** 2019-01-22

**Authors:** I. M. Shapey, A. Summers, T. Augustine, D. van Dellen

**Affiliations:** ^1^ Faculty of Biology, Medicine and Health University of Manchester Manchester UK; ^2^ Department of Renal and Pancreatic Transplantation, Manchester Royal Infirmary Manchester University NHS Foundation Trust Manchester UK

## Abstract

**Background:**

Normothermic regional perfusion (NRP) is a novel technique that aids organ recovery from donors after circulatory death (DCDs). However, ethical concerns exist regarding the potential return of spontaneous cerebral and cardiac activity (ROSCCA). This study aimed to determine the likelihood of ROSCCA in NRP‐DCDs of abdominal organs.

**Methods:**

Extracorporeal cardiopulmonary resuscitation (ECPR) for refractory out‐of‐hospital cardiac arrest (OOHCA) was identified as a comparator for NRP‐DCDs and as a validation cohort. A systematic search identified all articles relating to NRP‐DCDs and ECPR‐OOHCA. Rates of ROSCCA and survival outcomes (ECPR‐OOHCA only) were recorded and analysed according to the duration of no perfusion.

**Results:**

In NRP‐DCDs, 12 of 410 articles identified by database searching were eligible for inclusion. There were no instances of ROSCCA recorded among 493 donors. In ECPR‐OOHCA, eight of 947 screened articles were eligible for inclusion (254 patients). Where the absence of perfusion exceeded 5 min in ECPR‐OOHCA, there were no survivors with a favourable neurological outcome.

**Conclusion:**

ROSCCA is unlikely following commencement of NRP and has not occurred to date. Strict observance of the 5‐min interval following asystole provides satisfactory assurance that ROSCCA will not occur following NRP.

## Introduction

Normothermic regional perfusion (NRP) is a technique that aids organ recovery from donors after circulatory death (DCDs), leading to acceptable transplantation outcomes^1^. NRP was first used in 1989 by Spanish transplant surgeons using a percutaneously placed cardiopulmonary bypass circuit^2^. Autologous blood from the donor is used as a perfusate and is anticoagulated with heparin.

NRP closely resembles other extracorporeal membrane oxygenation (ECMO) circuits that can be used for cardiopulmonary bypass, extracorporeal cardiopulmonary resuscitation (ECPR) or for ventilatory assistance in patients with refractory acute lung injury. ECMO provides mechanical ventilatory and circulatory support via an extracorporeal circuit incorporating a membrane oxygenator, centrifugal pump or roller, heat exchanger and perfusate reservoir. NRP and ECMO have differences, which include the anatomical location of cannulas (peripheral *versus* central) and flow rates (lower in NRP).

NRP improves the viability of organs for kidney and liver transplantation, and leads to better post‐transplantation function with fewer complications^1^. Recently, NRP has been used to support donation of cardiothoracic organs, permitting recirculation and restoration of cardiac activity leading to transplantation^3^.

NRP works in three ways. First, it acts as a perfusion bridge between asystole and organ procurement, and allows organ procurement without further injury from prolonged ischaemia. Second, it enables rehabilitation at a cellular level by replenishing mitochondrial stores of adenosine 5′‐triphosphate, thereby mitigating against the effects of anaerobic metabolism and mimicking a period of ischaemic preconditioning^4^, ^5^, ^6^. Third, it permits donor organ assessment under non‐ischaemic conditions over a period of time, as well as the opportunity to track physiological responses to reperfusion.

A number of ethical considerations exist regarding the use of NRP. These include premortem cannulation and systemic heparinization; variable requirements for stand‐off time (the interval between confirmation of death and commencement of the perfusion process); and the potential for reperfusion to lead to return of spontaneous cerebral and cardiac activity (ROSCCA). The latter could invalidate the declaration of death, thereby threatening the fundamental tenet of deceased donation following cardiac arrest. ROSCCA can be assured categorically only by exclusion of the coronary and cerebral circulations from the NRP circuit. In abdominal organ donors, this is achieved by mechanical occlusion of the supracoeliac aorta most commonly using an inflated balloon, or by cross‐clamping. Nonetheless, the potential for ROSCCA to occur while using an inflated aortic occlusion balloon remains poorly understood and characterized. Concerns have been expressed that NRP, with or without inflation of an intra‐aortic occlusion balloon, provides an unsatisfactory assurance that ROSCCA would not occur^7^, ^8^.

This study aimed to determine the likelihood of ROSCCA in NRP‐DCDs of abdominal organs, and the impact an inflated intra‐aortic occlusion balloon may have on the likelihood of ROSCCA. The ethical implications of this increasingly used organ preservation technique in an era of donor organ shortage are also explored.

## Methods

### Validation cohort and definition of terms

A validation cohort of patients who most closely resembled organ donors, but who received ECMO for therapeutic indications without an inflated intra‐aortic occlusion balloon, was identified to determine the likelihood of ROSCCA occurring in NRP‐DCDs. Patients receiving ECMO as part of resuscitative measures following cardiac arrest (ECPR for out‐of‐hospital cardiac arrest (OOHCA)) were considered an appropriate comparator for NRP‐DCDs. This is because ECPR is employed for refractory cardiac arrest where cessation of resuscitative efforts would lead to the inevitable declaration of death.

The technical and mechanical components of NRP and ECPR are virtually identical, but two important distinctions exist. First, ECPR has resuscitative intent whereas the intent of NRP is preservation; and, second, there is no intra‐aortic occlusion balloon in ECPR.

In OOHCA, a variable period of absent perfusion (no‐flow time) exists between the onset of cardiac arrest and the commencement of cardiopulmonary resuscitation (CPR) with chest compressions, either by a bystander or a trained professional. A period of low flow exists from the commencement of mechanical compressive CPR until the return of perfusion by either return of spontaneous circulation or ECPR.

In the organ donation process, stand‐off time refers to the interval after declaration of death up to commencement of the donor perfusion and organ preservation process. A stand‐off time, commonly 5 min, is necessary in most jurisdictions to ensure that autoresuscitation does not take place and that the declaration of death is valid^9^. In uncontrolled DCDs there is, therefore, a no‐flow time, a low‐flow time and a stand‐off time before return of perfusion by NRP. In controlled DCDs there is only a low‐flow time (following withdrawal of life‐sustaining treatment) and a stand‐off time (following asystole). ECPR‐OOHCA is therefore an appropriate surrogate to determine the likelihood of ROSCCA in NRP‐DCDs.

### Search methods

A search of MEDLINE, Embase and the Cochrane Library electronic databases was performed to identify all articles relating to NRP for DCDs of abdominal organs. The following Medical Subject Heading (MeSH) terms were used and combined using Boolean operators: ‘extracorporeal membrane oxygenation’, ‘donors after cardiac death’, ‘non‐heart‐beating donors’, ‘donors after circulatory death’, ‘normothermic recirculation’, ‘normothermic perfusion’, ‘regional perfusion’, ‘liver transplantation’, ‘kidney transplantation’ and ‘pancreas transplantation’. A further search was carried out in an identical manner to identify all articles relating to ECPR‐OOHCA. The following MeSH terms were used: ‘extracorporeal life support’, ‘extracorporeal resuscitation’, ‘extracorporeal cardiopulmonary resuscitation’, ‘extracorporeal membrane oxygenation’ and ‘out‐of‐hospital cardiac arrest’. References of all identified papers were searched to ensure a comprehensive review. PRISMA guidelines were followed^10^. Ethical approval was not required.

### Inclusion and exclusion criteria

All study designs including cohort studies, case–control studies and case series were considered eligible. Studies performed between January 1997 and June 2016 were included. Case reports, conference abstracts, review articles, animal studies and articles not written in English were excluded. For the NRP‐DCD search, studies of organ donation of non‐abdominal organs and those using an *ex situ* perfusion process were also excluded. For the ECPR‐OOHCA search, articles that did not describe the relationship between no‐flow time and outcome were excluded. Publications reporting outcomes in ECPR for in‐hospital cardiac arrest were excluded, because the mean delay from cardiac arrest to commencement of CPR is much shorter than in OOHCA.

### Data extraction

Data extraction was undertaken using a standard pro forma. Patient and donor age, perfusion flow rates, and rates of failure in establishing successful perfusion were recorded. The NRP and ECPR protocols were interrogated, and exclusion and inclusion criteria identified and compared. Where recorded, methods of preventing and identifying cerebral and cardiac perfusion in NRP‐DCDs were also identified. In ECPR‐OOHCA, the duration of no flow was recorded.

### Outcome measures

Survival outcomes (discharge from hospital with a favourable neurological outcome) and cause of death following ECPR‐OOHCA were recorded and analysed according to the duration of absence of perfusion. Neurological outcomes were considered favourable if recorded as having a cerebral performance category of 1 or 2 (*Table* 1)^11^. In the NRP‐DCD groups, the incidence of ROSCCA was recorded. Survival following ECPR‐OOHCA, where ECPR commenced beyond the critical time of 5 min, was used as a surrogate marker to determine the likelihood of ROSCCA associated with NRP‐DCDs.

**Table 1:** Cerebral performance categories

Scale	Category	Level of function
1	Good cerebral performance	Normal living
2	Moderate cerebral disability	Sufficient function for independent activities of daily living
3	Severe cerebral disability	Limited cognition Fully dependent on others for daily living
4	Coma or vegetative state	Cerebral unresponsiveness or any degree of coma without fully meeting the criteria for brain death
5	Brain death	Apnoea, areflexia and electroencephalographic silence

These provide guidance regarding functional neurological recovery status following brain injury. Adapted from Safar^11^.

## Results

**Figure 1:**
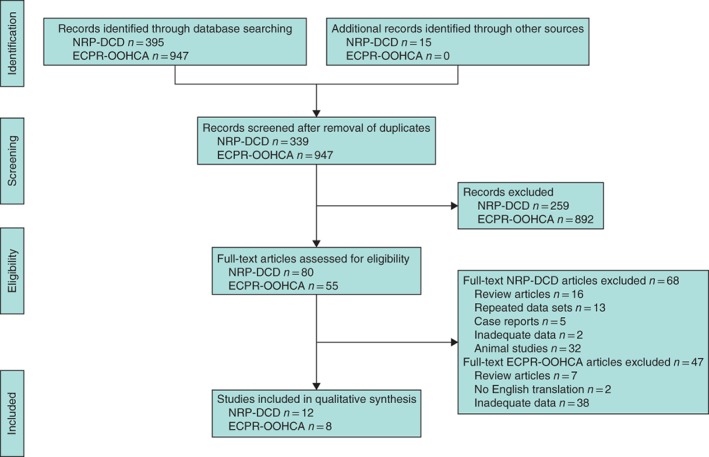
PRISMA flow diagram showing selection of articles for review. NRP‐DCD, normothermic regional perfusion for donors after circulatory death; ECPR‐OOHCA, extracorporeal resuscitation for out‐of‐hospital cardiac arrest

The database searches identified 410 articles relating to NRP‐DCDs, 12 of which were eligible for inclusion^12^, ^13^, ^14^, ^15^, ^16^, ^17^, ^18^, ^19^, ^20^, ^21^, ^22^, ^23^, and 947 articles relating to ECPR‐OOHCA, of which eight were eligible for inclusion^24^, ^25^, ^26^, ^27^, ^28^, ^29^, ^30^, ^31^ (*Fig*. 1). This provided a cumulative total of 493 potential DCDs who received NRP organ preservation and 254 patients who received ECPR following OOHCA.

### Protocols

The NRP‐DCD and ECPR‐OOHCA perfusion protocols are reported in *Tables* 2 and 3 respectively. NRP‐DCD protocols used an inflated occlusion balloon in the supracoeliac or thoracic aorta to prevent reperfusion of supradiaphragmatic organs in all but one series^13^. Vascular access was obtained via the femoral vessels in all but one case each of NRP and ECPR. Oniscu and colleagues^13^ performed rapid laparotomy, cannulation of the aorta and vena cava, and cross‐clamping of the thoracic aorta. There was no difference between NRP‐DCD and ECPR‐OOHCA eligibility criteria and cohorts with respect to patient and donor age, perfusion circuit flow rates, and rate of failure to commence the perfusion process.

**Table 2:** Perfusion protocols for normothermic regional perfusion donor after circulatory death programmes

Reference	Location	Donor type	Flow rate	Age eligibility (years)
Demiselle *et al*.^12^	France	Uncontrolled	2–3·7 l/min	18–60
Oniscu *et al*.^13^	UK	Controlled	1·7–4 l/min	Variable*
Fondevila *et al*.^14^	Spain	Uncontrolled	1·7 l/min	< 65
Jiménez‐Galanes *et al*.^15^	Spain	Uncontrolled	3·1 l/min	< 50
Otero *et al*.^16^	Spain	Uncontrolled	n.r.	< 50
Rojas‐Peña *et al*.^17^	USA	Controlled	> 45 ml per kg per min	0·5–65
Sánchez‐Fructuoso *et al*.^18^	Spain	Uncontrolled	n.r.	< 60
Valero *et al*.^19^	Spain	Uncontrolled	1–2 l/min	< 65
Reznik *et al*.^20^	Russia	Uncontrolled	2·5 l/min	n.r.
Farney *et al*.^21^	USA	Controlled	4–6 l/min	< 60
Lee *et al*.^22^	Taiwan	Controlled	2 l/min	n.r.
Koyama *et al*.^23^	Japan	Controlled	2–3·5 l/min	n.r.

* Depending on organ to be transplanted. n.r., Not reported.

**Table 3:** Perfusion protocol for extracorporeal cardiopulmonary resuscitation for out‐of‐hospital cardiac arrest programmes

Reference	Location	Commencement of ECPR in prehospital phase	Maximum no‐flow time (min)	Flow rate	Age eligibility (years)
Kagawa *et al*.^24^	Japan	No	< 15	> 2 l/min (target 2·5 l/min)	18–74
Bellezzo *et al*.^25^	USA	No	< 10	*S*vo_2_ > 70% MAP > 65 mmHg	n.r.
Ferrari *et al*.^26^	Germany	No	< 5	> 3 l/min	< 75
Maekawa *et al*.^27^	Japan	No	Variable	50–60 ml per min per kg	Variable
Lamhaut *et al*.^28^	France	Yes	< 5	2·5–4 l/min	< 70
Avalli *et al*.^29^	Italy	No	Variable	2·6 l per min per m^2^	12–75
Le Guen *et al*.^30^	France	No	< 5	3–4 l/min	n.r.
Mégarbane *et al*.^31^	France	No	Variable	2·5 l/min	n.r.

ECPR, extracorporeal cardiopulmonary resuscitation; *S*vo_2_, venous oxygen saturation; MAP, mean arterial pressure; n.r., not reported.

### Outcomes

Demographic data and outcomes from the NRP‐DCD and ECPR‐OHCA programmes are reported in *Tables* S1 and S2 (supporting information) respectively. There were no reported instances or evidence of reperfusion leading to ROSCCA in any NRP‐DCD programme. No organ donation procedures were abandoned owing to concerns relating to potential cardiac and cerebral reperfusion and ROSCCA. In the ECPR‐OOHCA programmes, there were no survivors with a favourable neurological outcome where the absence of perfusion lasted more than 5 min.

## Discussion

There is no evidence to suggest that ROSCCA may occur following the institution of NRP in DCDs where the proximal aorta has been occluded. Survival with a favourable neurological outcome following ECPR for refractory OOHCA does not occur when the no‐flow time exceeds 5 min. Following ECPR‐OOHCA where a no‐flow time was not specified, a small cohort of patients (8 of 254) survived to discharge, either in a persistent vegetative state or with significant neurological disability. It is not possible using the existing data to provide categorical assurance that ROSCCA would not occur in NRP‐DCDs in the absence of a proximally occluded aorta. The periods of no flow and low flow preceding the stand‐off time, in uncontrolled and controlled DCDs respectively, inevitably compound the ischaemic insult and further reduce the likelihood of ROSCCA.

Japan presents an interesting situation in that organ donation after brain death, although legal, is not acceptable culturally, but the country also has the largest worldwide practice of ECPR‐OOHCA^32^, ^33^. Studies by Morimura and colleagues^32^ and Sakamoto *et al*.^33^ were not eligible for inclusion in this review as the rates of survival and ROSCCA were not reported in relation to the no‐flow time or bystander CPR. Survivors of OOHCA with a favourable neurological outcome (12·3 per cent) and those who remained in a coma or vegetative state (10·4 per cent) were increased in equal proportion following use of ECPR, thereby providing scope for ethical debate on the overall impact of ECPR^33^. However, in Japan, with a paucity of deceased donor organs, public perception, as well as that of health policymakers, may be swayed in favour of ECPR‐OOHCA given that it could also lead to 60–70 additional organ donors annually.

Organ donation from DCDs is a sensitive issue, with associated fears of NRP‐related ROSCCA that are both appreciable and justified, especially given the reported incidents of prolonged survival after brain death^34^. However, such concerns are not supported by the existing evidence based on current practice. Non‐invasive methods of recording cerebral and coronary blood flow exist as an additional safety measure. A Doppler ultrasound probe can be placed over the carotid artery to detect cerebral blood flow and a radial artery catheter is inserted to detect pressure changes^35^. In Michigan, a lidocaine bolus (1–2 g) was included in the blood perfusate to prevent return of spontaneous cardiac activity^17^. If cardiac transplantation from NRP‐DCDs is to remain a regular feature of organ donation practice, monitoring of cerebral activity with an electroencephalogram may provide a necessary adjunct, as a safeguarding measure.

An inflated occlusion balloon in the proximal aorta has significant advantages, as perfusion can be commenced following a minimally invasive approach, and allows organ assessment before a definitive decision on organ procurement is made. In some instances, it is possible that the integrity of the inflated aortic occlusion balloon may have been compromised, but went unrecognized and unreported. However, balloon integrity can be confirmed by the presence/absence of a radial BP signal, both before and after commencing NRP. In the UK, cross‐clamping the thoracic aorta following rapid thoracolaparotomy is the preferred method for NRP‐assisted controlled DCD procurement of abdominal organs. However, thoracotomy ought not be a prerequisite for NRP, and minimally invasive NRP provides particular advantages in uncontrolled donors in societies where a presumed consent process is part of the legislation.

It has been mooted that NRP‐DCDs without a proximal occlusion of the aorta would be more appropriately termed as donors after brain circulatory death because recirculation of blood occurs following NRP, and that this procedure should therefore require specific consent^8^. The ethical and physiological boundaries of life and death remain controversial, and it is therefore imperative to characterize the impact of recirculation on the potential likelihood of ROSCCA as this clearly has significant implications for consent in organ donation^34^. The absence of ROSCCA may imply that no additional specific consent would be required.

In countries where ECPR‐OOHCA is practised routinely, the boundaries between ECPR for resuscitative purposes and NRP as an organ preservation technique may become blurred. Where a patient resuscitated by ECPR has a cerebral performance category of 4 or 5 but is dependent on ECMO for circulatory support, management of the potential organ donor should proceed identically to that for ECMO‐independent patients. However, ECMO‐independent patients existing in a vegetative state present a very challenging set of circumstances that requires careful individual consideration by families, clinicians and judicial representatives.

The main limitation of this study is the small number of ECPR‐OOHCA papers that were eligible for inclusion to approximate NRP‐DCD donation. Only eight of 947 potential papers on ECPR‐OOHCA were eligible and a proportion of articles, especially those from Japan, were excluded; this could have contributed to unintended bias. The criteria for the declaration of death, in particular brain death, remain controversial across the spectrum of clinical, scientific and religious communities, including within societies where organ donation practices are well established^34^.

This study has demonstrated that ROSCCA leading to survival with favourable neurological outcome following ECPR‐OOHCA does not occur when the duration of no flow exceeds 5 min. This suggests a similar process in NRP‐DCDs, with a stand‐off time of 5 min. ROSCCA in NRP‐DCDs with occlusion of the proximal aorta has not been reported to date. The presence of an occlusion balloon provides an additional mechanical level of assurance in a very sensitive clinical scenario. This is particularly important as any adverse events would have a catastrophic impact on the public perception of organ donation and transplantation.

## Supporting information


**Table S1** Demographics and outcomes from the normothermic donor after circulatory death (NRP‐DCD) programmes
**Table S2** Demographics and outcomes from the extracorporeal cardiopulmonary resuscitation for out of hospital cardiac arrest (ECPR‐OOHCA) programmes

## References

[1] Shapey IM , Muiesan P . Regional perfusion by extracorporeal membrane oxygenation of abdominal organs from donors after circulatory death: a systematic review. Liver Transpl 2013; 19: 1292–1303. 24136827 10.1002/lt.23771

[2] Sánchez‐Fructuoso AI , Prats D , Torrente J , Pérez‐Contín MJ , Fernández C , Alvarez J *et al* Renal transplantation from non‐heart beating donors: a promising alternative to enlarge the donor pool. J Am Soc Nephrol 2000; 11: 350–358. 10665943 10.1681/ASN.V112350

[3] Messer SJ , Axell RG , Colah S , White PA , Ryan M , Page AA *et al* Functional assessment and transplantation of the donor heart after circulatory death. J Heart Lung Transplant 2016; 35: 1443–1452. 27916176 10.1016/j.healun.2016.07.004

[4] Amador A , Grande L , Martí J , Deulofeu R , Miquel R , Solá A *et al* Ischemic pre‐conditioning in deceased donor liver transplantation: a prospective randomized clinical trial. Am J Transplant 2007; 7: 2180–2189. 17697262 10.1111/j.1600-6143.2007.01914.x

[5] Net M , Valero R , Almenara R , Barros P , Capdevila L , López‐Boado MA *et al* The effect of normothermic recirculation is mediated by ischemic preconditioning in NHBD liver transplantation. Am J Transplant 2005; 5: 2385–2392. 16162186 10.1111/j.1600-6143.2005.01052.x

[6] García‐Valdecasas JC , Tabet J , Valero R , Taurá P , Rull R , García F *et al* Liver conditioning after cardiac arrest: the use of normothermic recirculation in an experimental animal model. Transpl Int 1998; 11: 424–432. 9870271 10.1007/s001470050169

[7] British Transplantation Society and Intensive Care Society . *Organ Donation after Circulatory Death: Report of a Consensus Meeting*; 2010. https://nhsbtdbe.blob.core.windows.net/umbraco‐assets‐corp/1360/donation‐after‐circulatory‐death‐dcd_consensus_2010.pdf [accessed 31 October 2018].

[8] Dalle Ave AL , Shaw DM , Bernat JL . Ethical issues in the use of extracorporeal membrane oxygenation in controlled donation after circulatory determination of death. Am J Transplant 2016; 16: 2293–2299. 26999771 10.1111/ajt.13792

[9] NHS Blood and Transplant . *Management Process Description MPD880/6 Organ Retrieval: Pre‐Theatre DCD*; 2017. https://nhsbtdbe.blob.core.windows.net/umbraco-assets-corp/4939/organ_retrieval_-_pre_theatre_dcd_mpd880.pdf [accessed 5 April 2018].

[10] Moher D , Liberati A , Tetzlaff J , Altman DG ; PRISMA Group . Preferred reporting items for systematic reviews and meta‐analyses: the PRISMA statement. BMJ 2009; 339: b2535. 19622551 10.1136/bmj.b2535 PMC2714657

[11] Safar P . Resuscitation after brain ischemia In Brain Failure and Resuscitation, Grenvik A, Safar P (eds). Churchill Livingstone: New York, 1981; 155–184.

[12] Demiselle J , Augusto JF , Videcoq M , Legeard E , Dubé L , Templier F *et al* Transplantation of kidneys from uncontrolled donation after circulatory determination of death: comparison with brain death donors with or without extended criteria and impact of normothermic regional perfusion. Transpl Int 2016; 29: 432–442. 26606511 10.1111/tri.12722

[13] Oniscu GC , Randle LV , Muiesan P , Butler AJ , Currie IS , Perera MT *et al* *In situ* normothermic regional perfusion for controlled donation after circulatory death – the United Kingdom experience. Am J Transplant 2014; 14: 2846–2854. 25283987 10.1111/ajt.12927

[14] Fondevila C , Hessheimer AJ , Flores E , Ruiz A , Mestres N , Calatayud D *et al* Applicability and results of Maastricht type 2 donation after cardiac death liver transplantation. Am J Transplant 2012; 12: 162–170. 22070538 10.1111/j.1600-6143.2011.03834.x

[15] Jiménez‐Galanes S , Meneu‐Diaz MJ , Elola‐Olaso AM , Pérez‐Saborido B , Yiliam FS , Calvo AG *et al* Liver transplantation using uncontrolled non‐heart‐beating donors under normothermic extracorporeal membrane oxygenation. Liver Transpl 2009; 15: 1110–1118. 19718635 10.1002/lt.21867

[16] Otero A , Gómez‐Gutiérrez M , Suárez F , Arnal F , Fernández‐García A , Aguirrezabalaga J *et al* Liver transplantation from Maastricht category 2 non‐heart‐beating donors. Transplantation 2003; 76: 1068–1073. 14557754 10.1097/01.TP.0000085043.78445.53

[17] Rojas‐Peña A , Sall LE , Gravel MT , Cooley EG , Pelletier SJ , Bartlett RH *et al* Donation after circulatory determination of death: the University of Michigan experience with extracorporeal support. Transplantation 2014; 98: 328–334. 24825520 10.1097/TP.0000000000000070

[18] Sánchez‐Fructuoso AI , Marques M , Prats D , Conesa J , Calvo N , Pérez‐Contín MJ *et al* Victims of cardiac arrest occurring outside the hospital: a source of transplantable kidneys. Ann Intern Med 2006; 145: 157–164. 16880457 10.7326/0003-4819-145-3-200608010-00003

[19] Valero R , Cabrer C , Oppenheimer F , Trias E , Sánchez‐Ibáñez J , De Cabo FM *et al* Normothermic recirculation reduces primary graft dysfunction of kidneys obtained from non‐heart‐beating donors. Transpl Int 2000; 13: 303–310. 10959484 10.1007/s001470050706

[20] Reznik O , Skvortsov A , Loginov I , Ananyev A , Bagnenko S , Moysyuk Y . Kidney from uncontrolled donors after cardiac death with one hour warm ischemic time: resuscitation by extracorporal normothermic abdominal perfusion ‘*in situ*’ by leukocytes‐free oxygenated blood. Clin Transplant 2011; 25: 511–516. 20973824 10.1111/j.1399-0012.2010.01333.x

[21] Farney AC , Singh RP , Hines MH , Rogers J , Hartmann EL , Reeves‐Daniel A *et al* Experience in renal and extrarenal transplantation with donation after cardiac death donors with selective use of extracorporeal support. J Am Coll Surg 2008; 206: 1028–1037. 18471749 10.1016/j.jamcollsurg.2007.12.029

[22] Lee CY , Tsai MK , Ko WJ , Chang CJ , Hu RH , Chueh SC *et al* Expanding the donor pool: use of renal transplants from non‐heart‐beating donors supported with extracorporeal membrane oxygenation. Clin Transplant 2005; 19: 383–390. 15877803 10.1111/j.1399-0012.2005.00358.x

[23] Koyama I , Shinozuka N , Miyazawa M , Watanabe T . Total body cooling using cardiopulmonary bypass for procurement from non‐heart‐beating donors. Transplant Proc 2002; 34: 2602–2603. 12431540 10.1016/s0041-1345(02)03441-3

[24] Kagawa E , Inoue I , Kawagoe T , Ishihara M , Shimatani Y , Kurisu S *et al* Assessment of outcomes and differences between in‐ and out‐of‐hospital cardiac arrest patients treated with cardiopulmonary resuscitation using extracorporeal life support. Resuscitation 2010; 81: 968–973. 20627526 10.1016/j.resuscitation.2010.03.037

[25] Bellezzo JM , Shinar Z , Davis DP , Jaski BE , Chillcott S , Stahovich M *et al* Emergency physician‐initiated extracorporeal cardiopulmonary resuscitation. Resuscitation 2012; 83: 966–970. 22306260 10.1016/j.resuscitation.2012.01.027

[26] Ferrari M , Hekmat K , Jung C , Ferrari‐Kuehne K , Pfeifer R , Schlosser MH *et al* Better outcome after cardiopulmonary resuscitation using percutaneous emergency circulatory support in non‐coronary patients compared to those with myocardial infarction. Acute Card Care 2011; 13: 30–34. 21323411 10.3109/17482941.2010.542466

[27] Maekawa K , Tanno K , Hase M , Mori K , Asai Y . Extracorporeal cardiopulmonary resuscitation for patients with out‐of‐hospital cardiac arrest of cardiac origin: a propensity‐matched study and predictor analysis. Crit Care Med 2013; 41: 1186–1196. 23388518 10.1097/CCM.0b013e31827ca4c8

[28] Lamhaut L , Jouffroy R , Soldan M , Phillipe P , Deluze T , Jaffry M *et al* Safety and feasibility of prehospital extra corporeal life support implementation by non‐surgeons for out‐of‐hospital refractory cardiac arrest. Resuscitation 2013; 84: 1525–1529. 23827888 10.1016/j.resuscitation.2013.06.003

[29] Avalli L , Maggioni E , Formica F , Redaelli G , Migliari M , Scanziani M *et al* Favourable survival of in‐hospital compared to out‐of‐hospital refractory cardiac arrest patients treated with extracorporeal membrane oxygenation: an Italian tertiary care centre experience. Resuscitation 2012; 83: 579–583. 22056265 10.1016/j.resuscitation.2011.10.013

[30] Le Guen M , Nicolas‐Robin A , Carreira S , Raux M , Leprince P , Riou B *et al* Extracorporeal life support following out‐of‐hospital refractory cardiac arrest. Crit Care 2011; 15: R29. 21244674 10.1186/cc9976 PMC3222065

[31] Mégarbane B , Deye N , Aout M , Malissin I , Résière D , Haouache H *et al* Usefulness of routine laboratory parameters in the decision to treat refractory cardiac arrest with extracorporeal life support. Resuscitation 2011; 82: 1154–1161. 21641711 10.1016/j.resuscitation.2011.05.007

[32] Morimura N , Sakamoto T , Nagao K , Asai Y , Yokota H , Tahara Y *et al* Extracorporeal cardiopulmonary resuscitation for out‐of‐hospital cardiac arrest: a review of the Japanese literature. Resuscitation 2011; 82: 10–14. 20934798 10.1016/j.resuscitation.2010.08.032

[33] Sakamoto T , Morimura N , Nagao K , Asai Y , Yokota H , Nara S *et al*; SAVE-J Study Group. Extracorporeal cardiopulmonary resuscitation *versus* conventional cardiopulmonary resuscitation in adults with out‐of‐hospital cardiac arrest: a prospective observational study. Resuscitation 2014; 85: 762–768. 24530251 10.1016/j.resuscitation.2014.01.031

[34] Lewis A , Greer D . Current controversies in brain death determination. Nat Rev Neurol 2017; 13: 505–509. 28548107 10.1038/nrneurol.2017.72

[35] Currie I , Onisucu G , Reed M , Clegg G , McKeown D , Forsythe J . Organ Donation from the Emergency Department Category II DCD Pilot Programme Policy Document. NHS Blood and Transplant: Bristol, 2013.

