# Supplementary material for: Systematic review to assess the possibility of return of cerebral and cardiac activity after normothermic regional perfusion for donors after circulatory death
Source: Br J Surg. 2019 Jan 22;106(3):174–80. doi: 10.1002/bjs.11046 (PMC6749564; doi:10.1002/bjs.11046)
Supplement: Supplementary file 1 — Table S1 Demographics and outcomes from the normothermic donor after circulatory death (NRP‐DCD) programmes Table S2 Demographics and outcomes from the extracorporeal cardiopulmonary resuscitation for out of hospital cardiac arrest (ECPR‐OOHCA) programmes [file BJS-106-174-s001.docx]

**BJS11046**

**Systematic review to assess the possibility of return of cerebral and cardiac activity after normothermic regional perfusion for donors after circulatory death**

I. M. Shapey, A. Summers, T. Augustine and D. van Dellen

**Table S1** **Demographics and outcomes from the normothermic donor after circulatory death (NRP-DCD) programmes**

| **Lead author,**  **year of publication** | **Donors**  **(n)** | **Age**  **(years) *** | **ROSCCA**  **(n) **** |
| --- | --- | --- | --- |
| Demiselle 2016 [10] | 19 | 45.7 (5.7 SD) | 0 |
| Oniscu 2014 [11] | 21 | 46 (16-74) | 0 |
| Fondevila, 2012 [12] | 145 | 47 (27-56) | 0 |
| Jimenez-Galanes, 2009 [13] | 20 | 30.6 (18-46) | 0 |
| Otero, 2003 [14] | 14 | 23 (17-50) | 0 |
| Rojas Penna, 2014 [15] | 37 | 38.7 (9-65) | 0 |
| Sanchez-Fructuoso, 2006 [16] | 170# | 36.4 (11.5 SD) | 0 |
| Valero, 2000 [17] | 8 | 39.5 (15.6 SD) | 0 |
| Reznik, 2010 [18] | 10 | 44 (22-55) | 0 |
| Farney, 2008 [19] | 10 | NR | 0 |
| Lee, 2005 [20] | 16 | 38.6 (11.3 SD) | 0 |
| Koyama, 2002 [21] |  | 44.7 (17.3 SD) | 0 |

*Data are mean (range) unless stated ** incidence of evidence of potential ROSCCA

# 320 transplants reported

**Table S2** **Demographics and outcomes from the extracorporeal cardiopulmonary resuscitation for out of hospital cardiac arrest (ECPR-OOHCA) programmes**

| **Lead author,**  **year of publication** | **Age**  **(years) *** | **Patients**  **(n)** | **No-flow time (minutes) ***  **all patients** | **Survival**  **(n) #** | **No-flow time (minutes) ***  **survivors #** | **Survivors # (no-flow time > 5 minutes)** | **Cause of death** |
| --- | --- | --- | --- | --- | --- | --- | --- |
| Kagawa, 2010 [24] | 56 (49-64) | 39 | 1 (1-8) | 4 | 1 (0-5) | 0 | CC 26  MOF 3  HBI 4  Other 1$  CPC4 1$ |
| Bellezzo, 2012 [25] | 56 (13)  (Mean [SD]) | 8 | NR | 5 | 1 | 0 | Brain death 2  Other 1$ |
| Ferrari, 2011 [26] | 55 (NR) | 22 | NR | 6 | <5 | 0 | MOF 6  Cerebral oedema 5  CC 4  Bleeding 1 |
| Maekawa, 2013 [27] | 54 (47-60) | 53 | 2 (0-8) | 8 | 3 (0-4) | 0 | NR 39  CPC3/4 7$ |
| Lamhaut, 2013 [28] | 42 (16)  (Mean [SD]) | 6 | 4 (4)  (Mean [SD]) | 1 | <1 | 0 | Brain death 3  MOF 1 |
| Avalli, 2012 [29] | 46 (37-64) | 18 | 1(1-7) | 1 | <1 | 0 | Brain death 10  MOF 5  Other 2$$ |
| Le Guen, 2011 [30] | 42 (15)  (Mean [SD]) | 42 | 3 (1-6) | 2 | 1^st^ patient - 1  2^nd^ patient - <1 | 0 | Brain death 10  MOF 23  Haemorrhage 7 |
| Megarbane, 2011 [31] | 46 (39-55) | 66 | 2 (0-6) | 1 | 1 (0-5) | 0 | Brain death 3  HBI 12  Sepsis 3 |

NR = not reported; MOF = Multi-organ-failure; HBI = Hypoxic Brain Injury; CC = circulatory collapse *Data are median (range), unless stated; # denotes survival with favourable neurological outcome at discharge - Cerebral Performance Categories (CPC) 1 and 2; $ No cause stated; $$ Successfully weaned from ECMO but subsequently died, no cause stated
